# Prognostic Value of an Autophagy-Related Five-Gene Signature for Lower-Grade Glioma Patients

**DOI:** 10.3389/fonc.2021.644443

**Published:** 2021-03-09

**Authors:** Jin-Cheng Guo, Qing-Shuang Wei, Lei Dong, Shuang-Sang Fang, Feng Li, Yi Zhao

**Affiliations:** School of Traditional Chinese Medicine, Beijing University of Chinese Medicine, Beijing, China

**Keywords:** lower-grade glioma, autophagy related gene, prognostic biomarker, signature, survival

## Abstract

**Background:** Molecular characteristics can be good indicators of tumor prognosis and have been introduced into the classification of gliomas. The prognosis of patients with newly classified lower-grade gliomas (LGGs, including grade 2 and grade 3 gliomas) is highly heterogeneous, and new molecular markers are urgently needed.

**Methods:** Autophagy related genes (ATGs) were obtained from Human Autophagy Database (HADb). From the Cancer Genome Atlas (TCGA) and the Chinese Glioma Genome Atlas (CGGA), gene expression profiles including ATG expression information and patient clinical data were downloaded. Cox regression analysis, receiver operating characteristic (ROC) analysis, Kaplan–Meier analysis, random survival forest algorithm (RSFVH) and stratification analysis were performed.

**Results:** Through univariate Cox regression analysis, we found a total of 127 ATGs associated with the prognosis of LGG patients from TCGA dataset and a total of 131 survival-related ATGs from CGGA dataset. Using TCGA dataset as the training group (*n* = 524), we constructed a five-ATG signature (including BAG1, BID, MAP1LC3C, NRG3, PTK6), which could divide LGG patients into two risk groups with significantly different overall survival (Log Rank *P* < 0.001). Then we confirmed in the independent CGGA dataset that the five-ATG signature had the ability to predict prognosis (*n* = 431, Log Rank *P* < 0.001). We further discovered that the predictive ability of the five-ATG signature was better than the existing clinical indicators and IDH mutation status. In addition, the five-ATG signature could further classify patients after receiving radiotherapy or chemotherapy into groups with different prognosis.

**Conclusions:** We identified a five-ATG signature that could be a reliable prognostic marker and might be therapeutic targets for autophagy therapy for LGG patients.

## Introduction

Glioma is a malignant tumor that seriously threatens human health worldwide. It is characterized by complex histopathological types, large differences in patient prognosis, and limited treatment options. By convention, the histopathological classification (World Health Organization classification) divides patients with glioma into grades 1 to 4, and is widely used by clinicians to evaluate prognosis and guide treatment. However, due to the wide range of patients' survival within each grade, the evaluation results of the classic TNM classification are not entirely satisfactory. In recent years, with the in-depth research in molecular biology, scientists have found that IDH 1 and 2 mutations and 1p/19q codeletion play a more important role than TNM stage in the prognosis of glioma patients (1, 2). In 2016, the WHO made a major change in the classification of gliomas, incorporating molecular characteristics into the classification (3). Subsequently, in the field of glioma, deep mining of gene expression data and the discovery of more effective molecular markers have become research hotspots.

Lower-grade gliomas (LGGs, WHO grades II and III gliomas) are more likely to occur in young people aged 20–40 (4). LGGs are highly malignant and have a high mortality rate, which cause a huge burden on society, families and individuals. In the past decades, treatment methods such as surgery, radiotherapy and chemotherapy have continued to develop, and the survival rate of LGG patients has been greatly improved. However, the prognosis of patients is highly heterogeneous. Some patients survive for only 1 year, while others can survive for 15 years (5). Therefore, LGG currently is currently facing two major problems, that is, the molecular markers that have been discovered (such as IDH mutation and 1p/19q coding status) cannot completely accurately distinguish the prognosis of LGG, and the existing treatment methods cannot further improve the survival rate of patients.

Autophagy is a highly regulated catabolic process that can maintain cell homeostasis under basal and stress conditions. There is accumulating evidence showing that autophagy plays a complex and contradictory role in the occurrence and development of tumors, and has the dual effects of promoting and inhibiting tumorigenesis (6, 7). When gliomas are involved, the dual function of autophagy also appears. Shukla et al. found that ULK1/2 in glioblastoma was down-regulated, while overexpression of ULK2 increased autophagy and inhibited tumor cell growth (8). Pallichankandy et al. reported that the up-regulation of reactive oxygen species (ROS) in gliomas can activate the ERK1/2 pathway and trigger autophagic cell death (9). Therefore, some scientists believe that autophagy, as a tumor suppressor, can degrade damaged proteins and mitochondria, and prevent the activation of oncogenic signaling pathways and accumulation of p62 aggregates, thereby inhibiting the malignant behavior of tumors such as cell proliferation, migration and invasion (10). Since autophagy degradation produces a large number of amino acids, fatty acids and metabolic substrates, which provide abundant nutrients and convenient conditions for the growth of tumor cells, other researchers believe that autophagy plays a pro-tumoral role in gliomas (11). In short, autophagy is of great significance in gliomas. At present, new autophagy inducers or inhibitors that promote the autophagic death of glioma cells are being developed and are expected to become a new therapy for glioma (12–14).

Autophagy related genes (ATGs) are a group of evolutionarily highly conserved genes, which are essential molecules and participate in all stages of autophagy. In recent years, a growing number of ATGs and the etiological association between ATGs and tumors (15) have been discovered. Therefore, ATGs may have potential value in the prognosis of LGG patients. Our study aims to find the prognostic biomarkers of LGG through exploring the association between ATG and the survival of LGG patients and constructing prognostic gene signature.

## Materials and Methods

### Gene Expression Data and Clinical Characteristics of LGG Patients

The gene expression profile of patients with LGG in The Cancer Genome Atlas Program (TCGA, August 17, 2018) was tested experimentally using the Illumina HiSeq 2000 RNA Sequencing platform and obtained from the UCSC Xena browser (https://xenabrowser.net/). Another dataset was downloaded from Chinese Glioma Genome Atlas (CGGA) database (http://www.cgga.org.cn/). The BIGD accession number of LGG dataset in China National Genomics Data Center is PRJCA001747 (https://bigd.big.ac.cn/bioproject/browse/PRJCA001747). TCGA dataset was used as the training group, and CGGA dataset was used as test group. All clinical information of the LGG patients is shown in Table 1. In order to facilitate the subsequent data analysis, we discarded genes with missing expression values in more than 20% of LGG samples (16).

**Table 1:** Clinical characteristics of the LGG patients.

**Characteristic**	**TCGA (*n* = 524)**	**CGGA (*n* = 431)**
**Survival status**		
Living	387	242
Dead	137	189
**Age (years)**		
≤ 40	261	221
> 40	263	210
**Sex**		
Female	237	193
Male	287	238
**Grade**		
G2	257	180
G3	266	251
Unknown	1	
**IDH mutation status**		
Mutant	91	297
Wildtype	34	96
Unknown	399	38
**Chemo status**		
No	69	124
Yes	101	265
Unknown	354	42
**Radio status**		
No	173	86
Yes	284	314
Unknown	67	31

### Identification of Survival-Related Autophagy Related Genes in LGG

Autophagy related genes (ATGs) were obtained from the Human Autophagy Database (http://www.autophagy.lu/index.html), which contains the latest list of genes directly or indirectly involved in autophagy. We performed univariate Cox regression analysis to find the ATGs associated with patients' overall survival (OS) in the TCGA and CGGA datasets (*P* < 0.05).

### The Process of Constructing and Validating the Prognostic ATG Signature

In order to find an ideal prognostic signature, we constructed the prognostic signature with TCGA data, and then verified the predictive ability of the signature in independent CGGA data. Since there were a large number of survival-related ATGs in the TCGA data, we used the random survival forest algorithm (RSFVH) to reduce the number of ATGs. In addition, we used the ATGs selected above to perform Cox regression analysis and construct risk models as follows: $\text{Risk Score}=\sum_{\text{i}=1}^{\text{N}}\left(x_{i}{}^{*}Expression_{i}\right)$ where *N* is the number of ATG, *Expressioni* is the expression value of the ATG and ***x***_***i***_ is the coefficient of ATG in Cox regression analysis. Then, receiver operating characteristic (ROC) analysis was performed to compare the predictive power of different risk models. Finally, we used the ATG signature with the largest AUC value as the best prognostic signature (17).

After screening the best signature through the prognostic risk model, we performed survival analysis to test the predictive ability of the ATG signature in both the training and test groups.

### Statistical and Bioinformatics Analysis

Multivariable Cox regression analysis was performed to explore the predictive independence of the ATG signature. ROC was used to compare the survival prediction performance of the ATG signature with other prognostic markers. Kaplan–Meier analysis was tested to verify the stratification significance of the ATG signature for patients after receiving radiotherapy and chemotherapy (18). R 3.5.1 version (downloaded from www.r-project.org) with R packages including pROC, timeROC, randomForestSRC, and survival were used for analysis, where a *P*-value of < 0.05 was considered statistically significant.

## Results

### Identification of Survival-Related ATGs of LGG Patients

A total of 232 autophagy related genes were acquired from the HADb database. By comparison, we detected 208 ATGs from 26,440 expressed genes in the TCGA dataset (*n* = 524) and 210 ATGs from 23,998 expressed genes in the CGGA (*n* = 431) dataset. After analyzing the clinical data of 955 LGG patients, we found that the median age of the 955 LGG patients was 40 years (11–87 years), and there are more men in the affected population, indicating that LGG is more likely to occur in young adult males. In addition to gene expression data, patients also have complete radiotherapy, chemotherapy and IDH mutation status information, which is listed in Table 1.

**Figure 1:**
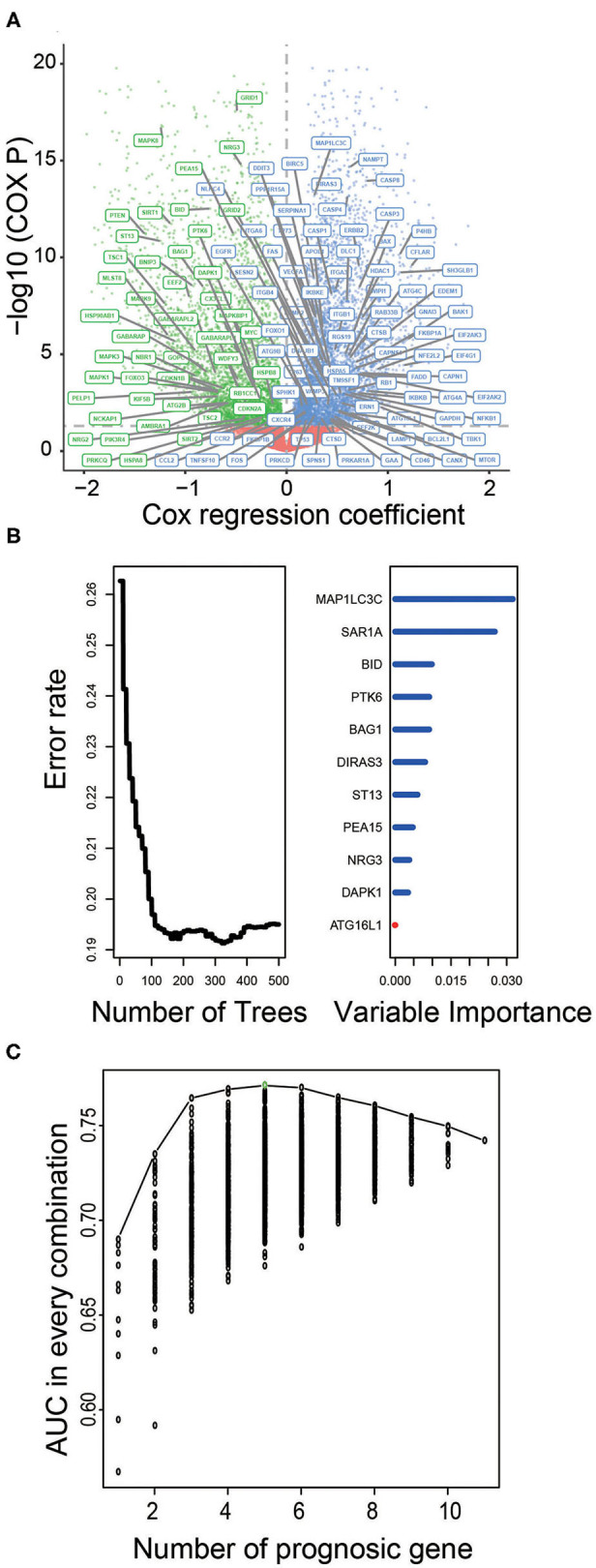
Identification of prognostic ATGs and constructing prognostic models in the training dataset. **(A)** Volcano plot showed the ATGs associated with LGG survival in the training set. **(B)** The prognostic ATGs were reduced to 11 by random forest supervised classification algorithm. **(C)** The prognostic five-ATG signature was selected for its largest AUC (AUC = 0.71).

To assess the prognostic significance of ATG in LGG, we performed univariate Cox regression analysis and identified 127 and 131 ATGs that were significantly associated with OS of LGG patients from the TCGA and CGGA datasets, respectively (*P* < 0.05, Figure 1A, Supplementary Table 1).

### Construction and Evaluation of Risk Prediction Model in the Training Dataset

We used the TCGA dataset as the training group to develop risk prediction model and construct prognostic signature. Since we discovered 127 ATGs associated with the survival of LGG patients (Figure 1A), we further performed RSFVH analysis and screened out 11 ATGs based on importance scores (Figure 1B). Then we used the 11 prognostic ATGs to develop the risk prediction model, and got 2^11^-1 = 2,047 ATG risk models. We performed ROC analysis and compared the predictive ability of 2,047 ATG risk models (Supplementary Table 2). The ATG signature including five ATGs (BAG1, BID, MAP1LC3C, NRG3, PTK6) was found to have the largest AUC value (AUC signature = 0.771; Figure 1C, Table 2). The selected risk model was calculated as follows: Risk score = (−0.75 × expression value of BID) + (−0.36 × expression value of PTK6) + (−0.82 × expression value of BAG1) + (−0.45 × expression value of NRG3) + (0.27 × expression value of MAP1LC3C). The regression coefficients of the four ATGs (BID, PTK6, BAG1, and NRG3) were all negative, which means they were genes related to poor prognosis, while MAP1LC3C was the opposite, indicating a good prognosis.

**Table 2:** The Prognostic significance of the ATGs in the signature in the TCGA dataset.

**Gene ID**	**HR**	**95% CI of HR**		**COX P**	**KM P**	**AUC**
		**Lower**	**Upper**			
BAG1	0.44	0.34	0.58	<0.001	<0.001	0.61
BID	0.47	0.38	0.58	<0.001	<0.001	0.61
NRG3	0.64	0.57	0.72	<0.001	<0.001	0.62
PTK6	0.70	0.59	0.82	<0.001	<0.001	0.62
MAP1LC3C	1.31	1.22	1.41	<0.001	<0.001	0.63

### Survival Prediction Performance of the Five-ATG Signature in the Training and Test Dataset

**Figure 2:**
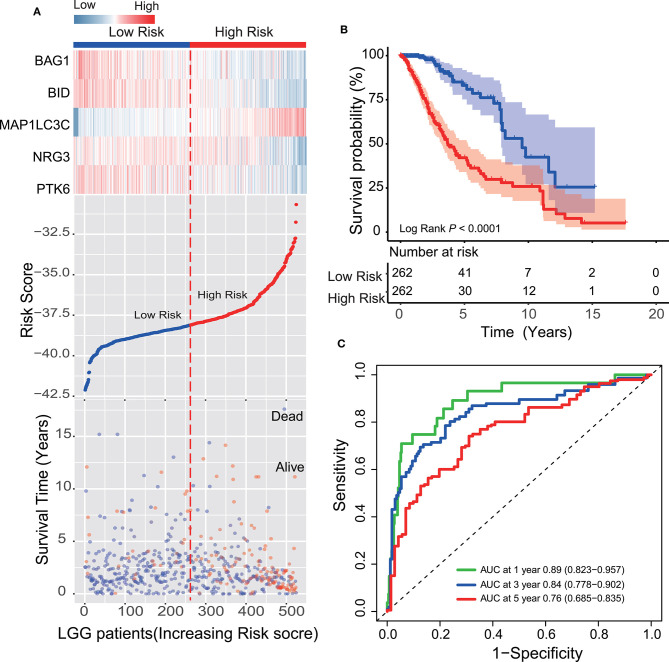
Survival prediction performance of the ATG signature in the training dataset. **(A)** Expression heatmap of the five ATGs, dot plot of risk scores and survival status of LGG patients in the training dataset. **(B)** Kaplan–Meier analysis demonstrated that the five-ATG signature divided patients into two risk groups with different survival. **(C)** Time-dependent ROC analysis of the ATG signature in the training group.

Each patient received a risk score based on the five-ATG signature. Then, the patients with LGG in the training dataset were divided into high-risk (*n* = 262) or low-risk group (*n* = 262) based on the median risk score. Through displaying the risk score, survival status and the five ATGs expression in a dot plot or heat map, we found that patients with high-risk scores had higher expression of BID, PTK6, BAG1, and NRG3 and were prone to death (Figure 2A). Kaplan–Meier analysis demonstrated that patients in the low-risk group owned longer survival times than those in the high-risk group (median survival time: 9.51 vs. 3.70 years, log-rank test *P* < 0.001; Figure 2B). Subsequently, we performed time-dependent ROC analysis to assess the prognostic accuracy of the five-ATG signature. In the TCGA dataset, the AUC for 1, 3, and 5 years of survival were 0.89, 0.84, and 0.76, respectively (Figure 2C).

**Figure 3:**
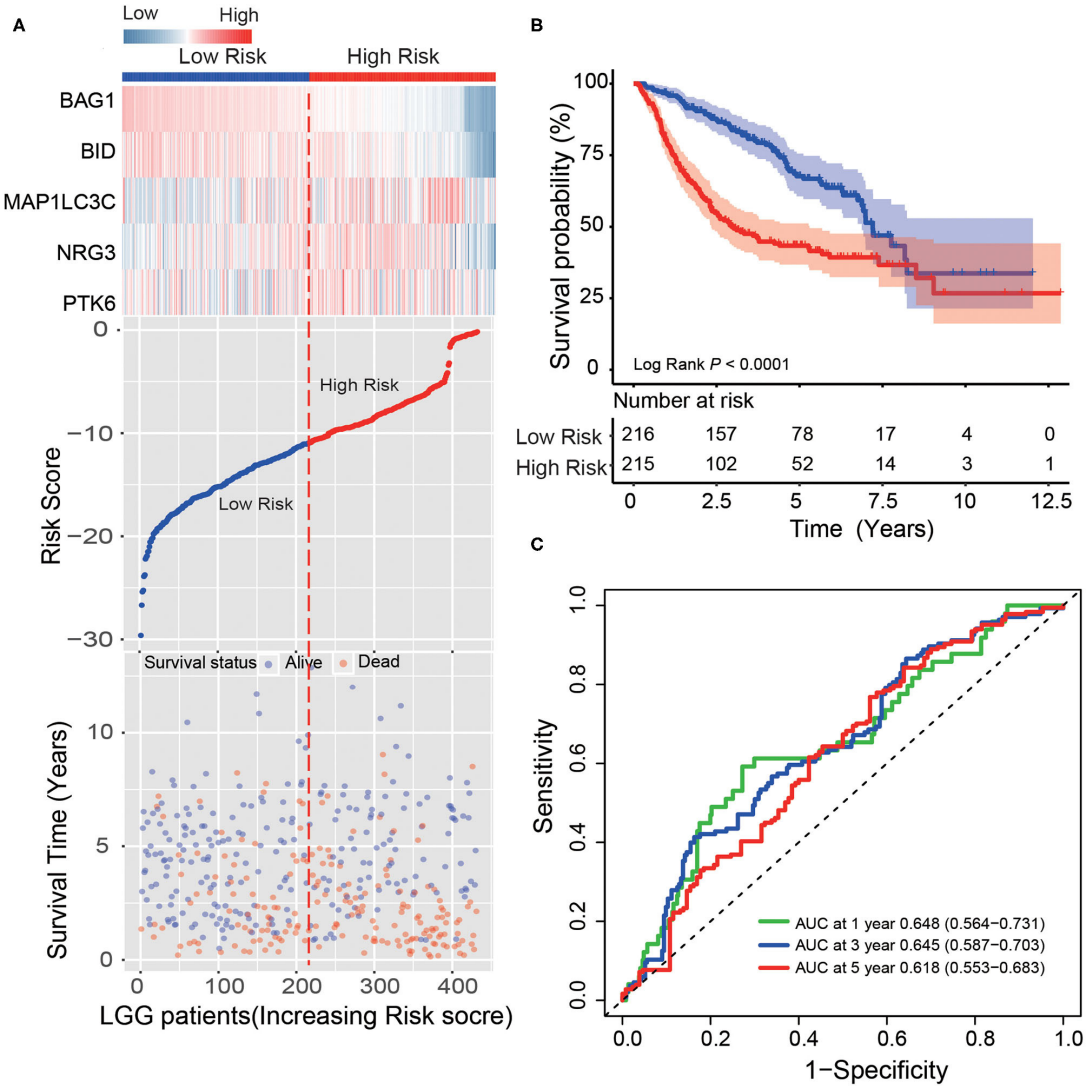
Validating survival prediction performance of the ATG signature in the test dataset. **(A)** Expression heatmap of the five ATGs, dot plot of risk scores and survival status of LGG patients in the test dataset. **(B)** Kaplan–Meier plot found that the five-ATG signature divided patients into high-risk and low-risk groups with different survival. **(C)** Time-dependent ROC analysis of the ATG signature in the test group.

In another large independent LGG dataset from CGGA (*n* = 431), the median risk score divided patients into high-risk or low-risk groups. From the dot plot and heat map, we found the relationship between the risk score, survival status and the five ATGs expression (Figure 3A). Kaplan–Meier analysis verified that the survival time of LGG patients in the high-risk group was shorter than that of patients in the low-risk group (median survival time: 2.96 vs. 7.21 years, log-rank test *P* < 0.001; Figure 3B). Time-dependent ROC analysis results showed that the AUC of the five-ATG signature was 0.65, 0.65, and 0.62 at the survival time of 1, 3, and 5 years in the CGGA dataset (Figure 3C).

### The Five-ATG Signature Is an Independent Predictive Factor

After confirming that the five-ATG signature has an excellent survival prediction ability, we tested its prognostic independence. We conducted Chi-square test in the training and test groups (*n* = 524/431) and found that the five-ATG signature was related to IDH mutation status and radiotherapy (Table 3). Then univariate and multivariable Cox regression analyses were conducted. The multivariable Cox regression results in the training and test datasets verified that the five-ATG signature can predict patients' survival without being affected by IDH mutation status and radiotherapy (High- vs. Low-risk, HR training = 8.71, 95% CI 1.1.86–40.71, *P* = 0.006, *n* = 524; HR test = 1.93, 95% CI 1.36–2.73, *P* < 0.001, *n* = 431, Table 4).

**Table 3:** Association of the ATG signature with clinical characteristics in LGG patients.

**Variables**	**TCGA group**		***P***	**CGGA group**		***P***
	Low risk^*^	High risk^*^		Low risk^*^	High risk^*^	
**Age (years)**			<0.001			0.53
≤ 40	155	106		107	114	
> 40	107	156		109	101	
**Sex**			0.22			0.88
Female	111	126		98	95	
Male	151	136		118	120	
**Grade**			<0.001			0.60
Unknown	0	1		0	0	
G2	171	86		87	93	
G3	91	175		129	122	
**IDH mutation status**			<0.001			<0.001
Unknown	188	211		34	4	
Mutant	64	27		157	140	
Wildtype	10	24		25	71	
**Radiotherapy**			<0.001			0.04
Unknown	31	36		22	9	
No	116	57		39	47	
Yes	115	169		155	159	
**Chemotherapy**			0.063			0.04
Unknown	187	167		29	13	
No	35	34		59	65	
Yes	40	61		128	137	

* *The median risk score divided patients into low risk group and high risk group*.

**Table 4:** Cox regression analysis of the signature with LGG survival.

			**Univariable analysis**				**Multivariable analysis**		
**Variables**		**HR**	**95% CI of HR**		***P***	**HR**	**95% CI of HR**		***P***
			**Lower**	**Upper**			**Lower**	**Upper**	
**TCGA dataset (*****n*** **=** **524)**									
Age	>40 vs. ≤ 40	2.82	1.96	4.04	<0.001	1.47	0.47	4.61	0.51
Sex	Male vs. female	1.14	0.81	1.60	0.45	2.03	0.70	5.84	0.19
IDH status	Wildtype vs. mutant	5.53	2.07	14.82	<0.001	3.64	1.20	11.06	0.02
LGG Grade	G3 vs. G2	3.31	2.28	4.79	<0.001	0.99	0.33	3.03	0.99
ATG-signature	High risk vs. low risk	4.33	2.87	6.53	<0.001	8.71	1.86	40.71	0.01
**CGGA set (*****n*** **=** **431)**									
Age	>40 vs. ≤ 40	1.19	0.89	1.58	0.24	1.13	0.84	1.52	0.42
Sex	Male vs. female	1.00	0.75	1.34	0.98	1.07	0.79	1.45	0.65
IDH status	Wildtype vs. mutant	2.24	1.64	3.07	<0.001	1.64	1.15	2.35	0.01
LGG Grade	G3 vs. G2	2.62	1.89	3.64	<0.001	2.97	2.10	4.20	<0.001
ATG-signature	High risk vs. low risk	2.31	1.72	3.11	<0.001	1.93	1.36	2.73	<0.001

### The Five-ATG Signature Is Better Than Existing Prognostic Indicators

**Figure 4:**
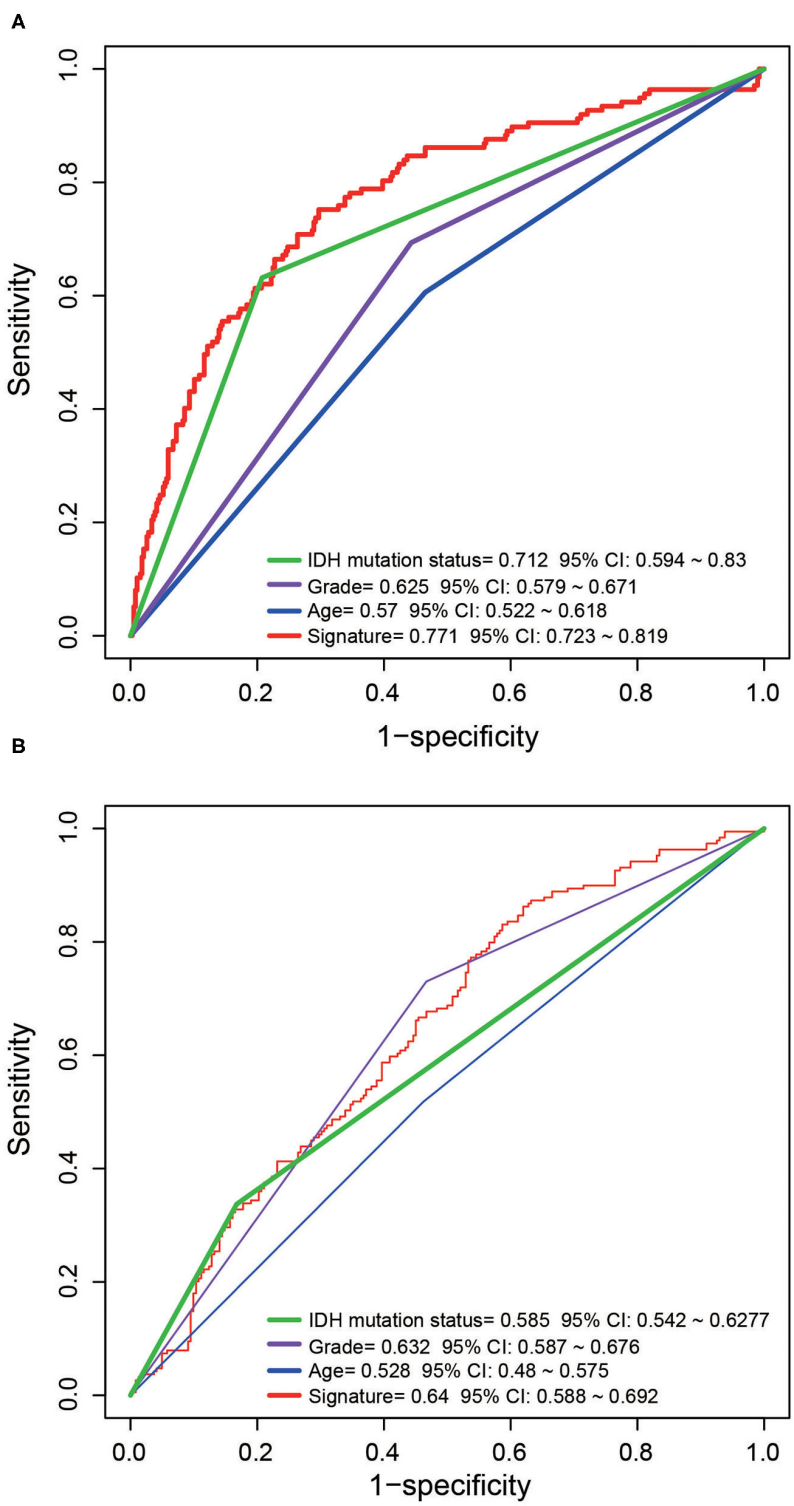
The predictive performance of the five-ATG signature was better than that of grade. Age and IDH mutation in the training **(A)** and test **(B)** datasets.

The prognostic indicators currently used clinically include age, TNM staging and IDH mutation status. To compare the predictive performance of the five-ATG signature with that of the existing indicators. We drew ROC curves and found that the five-ATG signature had the biggest AUC value in the training/test datasets (AUC signature 0.771/0.64; AUCIDH 0.712/0.585; AUC grade 0.625/0.632; AUC age 0.57/0.528, Figure 4), indicating that the five-ATG signature had a better survival prediction performance.

### Radiotherapy and Chemotherapy Stratification Analysis

**Figure 5:**
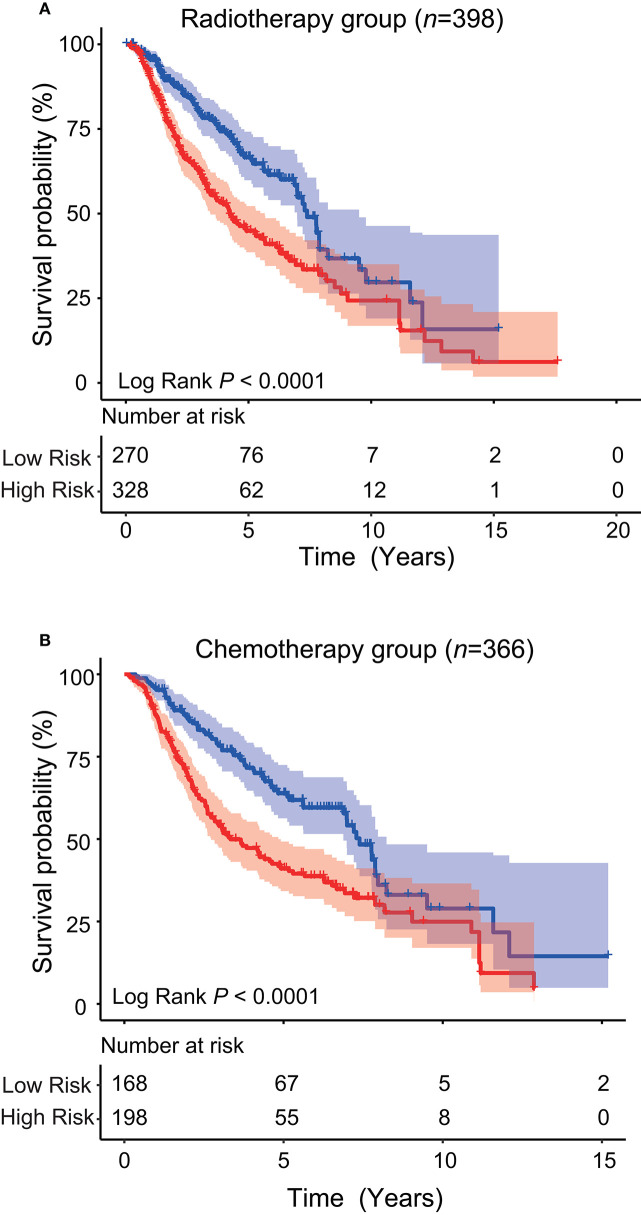
Stratification analysis. The five-ATG signature could further divide patients after radiotherapy **(A)** or patients after chemotherapy **(B)** into two groups with significantly different survival.

Since LGG is milder than the highest-grade GBM, it is controversial whether LGG patients should receive radiotherapy or chemotherapy immediately after diagnosis, or “wait and see.” For those who have received treatment (radiotherapy or chemotherapy), whether this five-ATG signature can predict prognosis is worth exploring. After determining the prognostic value of the signature, we conducted stratification analysis of all patients from TCGA and CGGA who received radiotherapy or chemotherapy. From Table 1, we obtained a total of 598 cases who received radiotherapy (284 from TCGA dataset and 314 from CGGA dataset). We observed these 598 patients who have undergone radiotherapy and found that these patients could be further divided into low- and high-risk groups with significantly different survival by the five-ATG signature (5 or 10-years survival: 68.47%/28.61 vs. 42.91%/18.71%, log-rank test *P* < 0.001, Figure 5A). Among the 559 patients with chemotherapy information in the two datasets, we found that a total of 366 patients received chemotherapy. Figure 5B showed that the five-ATG signature divided these 559 patients into two groups with different prognosis (5 or 10-years survival: 63.62%/33.44 vs. 42.56%/22.29%, log-rank test *P* < 0.001, Figure 5B).

## Discussion

After the new molecular classification of glioma in 2016, LGG has become a large group of tumors that is different from glioblastoma and grade I glioma in terms of prognosis. However, biological tumor heterogeneity is still a huge challenge for patient prognosis assessment and precise treatment, which makes the prognosis, treatment response and drug resistance of patients vary greatly. Recently, the exploration of the molecular characteristics of glioma has provided many potential markers that can be used for glioma classification, prognosis judgment and treatment guidance. In the present study, we analyzed gene expression and clinical data of 955 LGG cases from two different cohorts, and developed a prognostic signature based on the expression of autophagy related genes.

Due to its invasive growth characteristics, LGG cannot be completely removed by surgery, nor can be cured by existing treatment options, and gradually turns into a lethal glioblastoma. Therefore, it is justified to find new prognostic biomarkers and therapeutic strategies. In recent years, with the development of related research on the role of autophagy in tumors, researchers have discovered that ATGs could be used as prognostic biomarkers and be potential therapeutic targets. Among them, Beclin-1, and microtubule-associated protein light chain 3 (LC3) have become research hotspots. Studies have shown that abberrant expression of Beclin-1 is strongly associated with the poor survival of various tumors such as intrahepatic cholangiocarcinoma (19), endometrial adenocarcinoma (20), colorectal cancer (21). LC3 was found to be related to tumor growth and progression of triple negative breast cancer (22), and be associated with clinical prognosis of pancreatic cancer (23), hepatocellular carcinoma (24) and colorectal cancer (25). Our study found that 127 and 131 ATGs were closely related to the OS of LGG patients from the TCGA and CGGA datasets, among which a total of 80 ATGs were present in both groups. After constructing the risk model, we screened out the signature composed of five ATGs (BID, BAG1, PTK6, NRG3, MAP1LC3C) with the best prognosis prediction performance, indicating these five ATGs play an important role in glioma.

BAG1 (Bcl-2-associated athanogene) and BID (BH3 interacting domain death agonist) are key genes that play a regulatory role in the process of apoptosis. BAG1 is an oncogene that can enhance the anti-apoptotic effect of BLC-2 (26) and is aberrantly expressed in multiple cancer types. It has been suggested that BAG1 can be used as a potential drug target (27). BID is a member of the BCL-2 family and promotes apoptosis (28). Studies have found that BID is associated with the survival of thyroid cancer and clear-cell renal cell carcinoma. PTK6 (Protein tyrosine kinase six) is a cytoplasmic non-receptor protein kinase thatis highly expressed in tumors such as breast cancer, bladder cancer, lung cancer, and ovarian carcinoma. It has been detected that PTK6 overexpression is correlate with the poor prognosis of bladder cancer (29), prostate cancer (30), and breast cancer (31). NRG3 (neuregulin three) is abundantly expressed in brain tissue. NRG3 has been demonstrated to be involved in oligodendrocyte survival through binding and activating erbB4 (32). This study found that BID, BAG1, PTK6, NRG3 were significantly related to the poor prognosis of LGG through gene expression profile data analysis, supplementing the possible role of these genes in LGG. MAP1LC3C (microtubule associated protein one light chain three gamma) is a member of ATG8 family and plays a critical role in the process of autophagy. In addition, studies have shown that cancer cells with low MAP1LC3C levels exhibit enhanced cell invasion. Consistent with this finding, we found that LC3C is an indicator of good clinical prognosis for LGG.

In recent years, gene signatures have been proven to be good molecular biomarkers for various types of cancer due to their powerful ability to distinguish the prognosis of cancer patients (33). In combination with the important role of autophagy in tumors, researchers are currently exploring the effectiveness of ATG-gene signature in evaluating tumor prognosis. Gu et al. found an autophagy-related prognostic signature for breast cancer (34). Zhou et al. developed an ATG-signature which could predict the post-operative survival of colorectal cancer patients (35). Yue et al. identified an ATG-signature that can be used to analyze the prognosis of pancreatic adenocarcinoma patients (36). In gliomas, Wang et al. constructed a signature with four autophagy-related genes and validated its prognostic performance in GBM; Xu et al. found an autophagy-related signature that can divided patients into different survival outcomes groups (37). For LGG patients, only one seven-ATG signature was constructed for individualized survival prediction (38). Our study constructed a five-ATG signature which had a good predictive performance and could be an independent prognostic factor. Moreover, we found the significance of the five-ATG signature in guiding the prognosis of LGG patients after radiotherapy or chemotherapy.

## Conclusion

In conclusion, our study found multiple ATGs related to the survival of LGG and developed a five-ATG signature based on the risk score model, which could be a promising prognostic biomarker for LGG patients. Further research on these five ATGs may contribute to provide targets for LGG autophagy therapy.

## Data Availability Statement

The original contributions presented in the study are included in the article/Supplementary Material, further inquiries can be directed to the corresponding author/s.

## Author Contributions

J-CG: data collection, data analysis, interpretation, and drafting. FL and YZ: study design, study supervision, and final approval of the manuscript. Q-SW, LD, and S-SF: technical support and critical revision of the manuscript. All authors read and approved the final manuscript.

## Conflict of Interest

The authors declare that the research was conducted in the absence of any commercial or financial relationships that could be construed as a potential conflict of interest.

## Abbreviations

LGG: lower-grade glioma
ATG: Autophagy related gene
TCGA: The Cancer Genome Atlas
CGGA: the Chinese Glioma Genome Atlas
ROC: receiver operating characteristic
KM: Kaplan–Meier
AUC: area under the ROC curve
OS: overall survival.
